# Cascaded exciton energy transfer in a monolayer semiconductor lateral heterostructure assisted by surface plasmon polariton

**DOI:** 10.1038/s41467-017-00048-y

**Published:** 2017-06-26

**Authors:** Jinwei Shi, Meng-Hsien Lin, I-Tung Chen, Nasim Mohammadi Estakhri, Xin-Quan Zhang, Yanrong Wang, Hung-Ying Chen, Chun-An Chen, Chih-Kang Shih, Andrea Alù, Xiaoqin Li, Yi-Hsien Lee, Shangjr Gwo

**Affiliations:** 1 Department of Physics, National Tsing-Hua University, Hsinchu, 30013 Taiwan; 2 Department of Physics and Applied Optics Beijing Area Major Laboratory, Beijing Normal University, Beijing, 100875 China; 3 Department of Physics, The University of Texas at Austin, Austin, Texas 78712 USA; 4 Department of Materials Science and Engineering, National Tsing-Hua University, Hsinchu, 30013 Taiwan; 5 Department of Electrical and Systems Engineering, University of Pennsylvania, Philadelphia, Pennsylvania 19104 USA; 6 Department of Electrical and Computer Engineering, The University of Texas at Austin, Austin, Texas 78712 USA; 7 National Synchrotron Radiation Research Center, Hsinchu, 30076 Taiwan

## Abstract

Atomically thin lateral heterostructures based on transition metal dichalcogenides have recently been demonstrated. In monolayer transition metal dichalcogenides, exciton energy transfer is typically limited to a short range (~1 μm), and additional losses may be incurred at the interfacial regions of a lateral heterostructure. To overcome these challenges, here we experimentally implement a planar metal-oxide-semiconductor structure by placing a WS_2_/MoS_2_ monolayer heterostructure on top of an Al_2_O_3_-capped Ag single-crystalline plate. We find that the exciton energy transfer range can be extended to tens of microns in the hybrid structure mediated by an exciton-surface plasmon polariton–exciton conversion mechanism, allowing cascaded exciton energy transfer from one transition metal dichalcogenides region supporting high-energy exciton resonance to a different transition metal dichalcogenides region in the lateral heterostructure with low-energy exciton resonance. The realized planar hybrid structure combines two-dimensional light-emitting materials with planar plasmonic waveguides and offers great potential for developing integrated photonic and plasmonic devices.

## Abstract (web summary)

Exciton energy transfer in monolayer transition metal dichalcogenides is limited to short distances. Here, Shi et al. fabricate a planar metal-oxide-semiconductor structure and show that exciton energy transfer can be extended to tens of microns, mediated by an exciton-surface-plasmon-polariton–exciton conversion mechanism.

## Introduction

Materials in the family of transition metal dichalcogenides (TMDs) form planar honeycomb lattices with closely matched lattice constants, making it possible to grow monolayer-thick lateral heterostructures (LHSs) using chemical vapor deposition (CVD)^1–5^. Consequently, novel device applications can be envisioned based on LHSs or lateral superlattices consisting of adjacent regions of materials with dissimilar band gaps. Following interband excitations, excitons (electron-hole pairs) with exceptionally large binding energy (e.g., for WS_2_, 320 meV^6^ are formed, making them stable even at room temperature and are relevant for device applications. For device applications based on two-dimensional (2D) TMDs, excitons created via local optical or electrical excitation in one TMD material needs to be transferred to different TMD regions in the LHS to activate the entire active material. Cascaded exciton energy transfer in LHS from one TMD region to another could enable flexible devices, taking advantage of the versatile lateral band structure engineering. Such a cascaded exciton energy transfer process is critical for optoelectronic devices (e.g., displays^7^, photovoltaics^8^ and cascaded fluorescence cameras^9^), which are usually based on a variety of materials such as multi-colour colloidal quantum dots and polymer films. However, several challenges exist in effective exciton energy transfer in TMDs. Importantly, exciton diffusion is typically limited to a rather short range (~1 μm) due to ultrafast exciton recombination lifetime and moderate exciton mobility in TMDs^10–14^. Furthermore, one dimensional (1D) confinement potential may form at the heterojunction of an LHS due to band bending^3, 15, 16^, causing exciton trapping and additional energy loss at the interface.

Here, we adopt a 2D metal-oxide-semiconductor (MOS) hybrid material platform to address the challenges of long-range exciton energy transfer on a TMD LHS. Our MOS structure consists of a WS_2_/MoS_2_ LHS transferred to a single-crystalline plasmonic plate (Ag plate) covered with a few nanometer thick Al_2_O_3_ layer^17^. The large optical density of states in the plasmonic structures allows the excitonic energy to be effectively transferred to surface plasmon polaritons (SPPs), which can propagate more than 100 μm in the visible frequency range^17^, facilitating long-range exciton energy transfer in TMD LHS. Specifically, we demonstrate that this planar MOS structure supports isotropic energy transfer with a propagation length ~40 μm, which is two orders of magnitude longer than the exciton diffusion length in bare TMD monolayers. Despite the large absorption coefficient of monolayer TMD at the exciton resonance, this propagation length is only about a factor of two shorter than the SPP propagation distance without the TMD layer, as confirmed by simulations. Furthermore, we show that the energy loss at the interface can be minimized in high-quality LHSs with atomically sharp interfaces. Our work suggests that a hybrid photonic/plasmonic material platform can leverage the advantages of both components.The TMD layers act as efficient light absorbers and emitters due to the concentrated, large oscillator strength associated with the exciton resonance^18^. However, the ultrafast exciton recombination dynamics^12–14^ seriously restrict the exciton energy transfer distance. Here, this limitation is circumvented by coupling the TMD layer to a low-loss plasmonic substrate, which supports waveguiding and energy transfer via SPPs over a sufficiently long distance and offers additional benefits such as the capability of shaping and enhancing the near field^19–21^. In contrast to the previously reported exciton-SPP or SPP-exciton conversion in 1D hybrid structures consisting of Ag nanowires and monolayer TMDs^22, 23^, the demonstrated long-distance exciton energy transfer process contains a complete cascaded exciton–SPP–exciton conversion in a fully planar geometry. This hybrid material platform offers promising perspectives in future integrated photonic/plasmonic applications by merging plasmonics with atomically thin semiconductors and their unique heterostructures^24–26^.

## Results

### Exciton energy transfer assisted by SPP

**Fig. 1:**
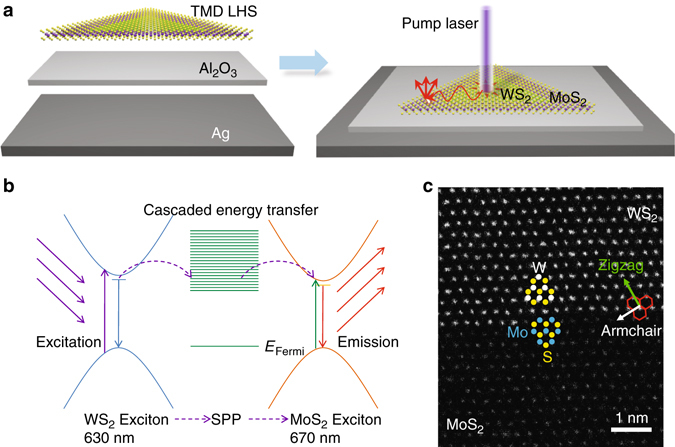
Schematic illustrations of the metal-oxide-semiconductor (MOS) structure and the cascaded exciton energy transfer process assisted by surface plasmon polariton. **a** A colloidal Ag plate is coated with ~3 nm Al_2_O_3_ immediately after it is taken out of the solution. The as-grown monolayer-thick WS_2_/MoS_2_ lateral heterostructure (LHS) is transferred to the Ag plate using a polymethyl methacrylate (PMMA)-assisted transfer technique. An excitation laser is normally incident on the WS_2_ region, and the photoluminescence (PL) from the interface or a specified MoS_2_ region is detected. **b** Energy transfer diagram describing the exciton–SPP–exciton conversion process. **c** High-resolution annular dark-field scanning transmission electron microscopy (STEM) image of a monolayer LHS grown with an atomically sharp interface

In Fig. 1a, we show a conceptual schematic of the energy transfer process in the MOS structure. An above-bandgap laser excitation (405 or 532 nm) is normally incident on the wide-gap WS_2_ region and creates excitons with photon emission energy near ~630 nm, as illustrated in Fig. 1b. The excitons then excite SPPs in the Ag plate via near-field, non-radiative energy transfer. The natural 2D geometric match between the atomically thin LHS and the plasmonic plate confines the excitonic energy in the 2D MOS structure and allows energy carried by SPPs to propagate on the planar waveguide. In the narrow-gap MoS_2_ region, the SPP is out-coupled at specific sites,which are created through intentionally introduced disruptions to the 2D structure, e.g., slots created via atomic force microscope (AFM) lithography or SiO_2_ spheres. The near-field of SPPs at the out-coupled site then locally excites MoS_2_ excitons (~670 nm), completing the exciton–SPP–exciton conversion process. The energy transfer occurs in a cascaded fashion involving energy transfer from a wide-gap region to a narrow-gap region in the LHS.

In this work, the material quality of the planar MOS photonic/plasmonic material platform is crucial for the successful demonstration of long-distance energy transfer. The TMD LHS typically consists of two concentric triangular regions. Depending on the particular CVD procedures used, the WS_2_/MoS_2_ interface conditions can be drastically different (see Methods for details). A one-step growth procedure^1^ produces as-synthesized WS_2_/MoS_2_LHS with inner triangles comprising of MoS_2._ The heterojunction of this WS_2_/MoS_2_ LHS displays an ideal 1D interface along the zigzag direction with an atomically sharp interface without compositional fluctuations or alloyed phases, as shown in the scanning transmission electron microscopy (STEM) image (Fig. 1c). This high-quality LHS enables energy transfer across the interface without measurable loss at the interfacial region. In contrast, a two-step growth procedure yields LHS (inner triangle: WS_2_) with rough interfacial regions decorated with stacked layers of small MoS_2_ flakes, as shown in Supplementary Fig. 1 and Note 1. The significant refractive-index change at the interface of the LHS leads to a visible energy loss, as demonstrated below. The Ag plates were grown using a room temperature wet-chemical synthesis method recently developed by us^17^. A few nanometer thick, atomically smooth Al_2_O_3_ layer was immediately grown by using the atomic layer deposition (ALD) technique on top of the Ag plate after removal from the colloidal solution. This oxide layer not only prevents the Ag plate from oxidation/degradation in the ambient environment, but also creates a dielectric spacer to reduce possible photoluminescence (PL) quenching resulting from non-radiative energy transfer between the TMD monolayer and the Ag plate.

**Fig. 2:**
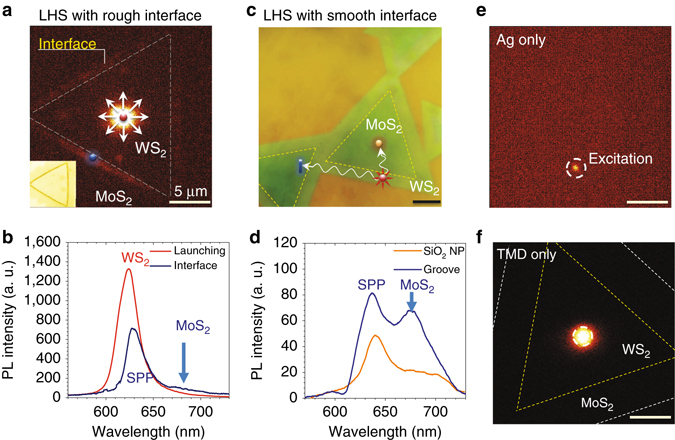
Experimental demonstration of SPP-assisted exciton energy transfer in a WS_2_/MoS_2_ TMD LHS. **a** PL spatial mapping from a metal-oxide-semiconductor (MOS) structure in which the LHS has rough interfaces. Excitons in WS_2_ (indicated by the *red dot*) created in the centre of the inner triangle are converted to SPPs, which propagate isotropically and are out-coupled at the interface, exciting the excitons from the MoS_2_ region (indicated by the *blue dot*). **b** PL spectra collected at the launching site and at the interface of the LHS shown in **a**. **c** An optical microscopy image of monolayer thick MoS_2_/WS_2_ LHS with atomically sharp interfaces. The laser excitation site (indicated by the *red star*), the out-coupling sites at a SiO_2_nanosphere (indicated by the *orange dot*), and a groove on the Ag plate (indicated by the *blue slit*) are labeled on the image. **d** The spectra taken at the out-coupling sites of the nanosphere and the groove, respectively. **e**, **f** The PL spatial mapping images obtained from an Ag plate alone **e**, and from a TMD LHS on a Si/SiO_2_ substrate **f**. The scale bars in all figures correspond to 5 µm

First, we examined the excitonic energy transfer process in an LHS with a rough interface, as shown in the inset of Fig. 2a. Excitons optically created in the inner triangular WS_2_ region act as exciton point sources and excite the SPP modes bound to the MOS structure. The excited SPPs propagate away from the launching site isotropically and are out-coupled at all triangular interfaces due to the significant change in effective refractive index in the rough interfacial region. This process is visually demonstrated in Fig. 2a. A spatial map of the PL signal from the entire TMD heterostructure was collected (see Methods) while the excitation laser was fixed at the centre of the WS_2_ triangle. The PL spectra collected from both the launching site (*red curve*) and collection site (*blue curve*) near the interface of the LHS are shown in Fig. 2b. In the spectrum collected from the rough interfacial region, the resonance near ~630 nm displays a small red shift in comparison to the spectrum collected at the launching site. This peak likely arises from directly scattered SPPs, which exhibit lower loss at longer wavelength. A weaker peak near 670 nm (indicated by the *blue arrow*) is observed in the spectra at collection sites near the interface. This peak coincides well with the excitonic energy in MoS_2_. This experiment demonstrates the possibility of cascaded excitonic energy transfer assisted by SPPs in TMD-based hybrid materials.

We then investigated the LHS with an atomically sharp interface. In this case, following the exciton–SPP excitation in the WS_2_ region at the outer triangle, the SPPs propagate through the interface region with little scattering loss because of the small difference in the refractive indices between two TMD regions. In order to observe the second step in the energy conversion process (i.e., SPP to exciton), either a nanogroove patterned by AFM lithography or a SiO_2_ nanosphere of 500 nm diameter placed at the selected out-coupling site by using AFM manipulation (illustrated in Fig. 2c) was utilized. Both methods out-couple SPPs, which can also excite the excitons in MoS_2_ either due to translational symmetry breaking or local hot SPP spots. Therefore, the spectra at the collection sites (*orange dot and blue groove*) feature a resonance near 630 nm dominated by scattered SPPs excited by excitons in the WS_2_ region and the exciton resonance in MoS_2_ near 670 nm excited by the out-coupled SPPs, as shown in Fig. 2d. These experimental results demonstrate that SPP-assisted exciton energy transfer from one to the other region of the TMD LHS occurs with a minimal scattering loss at the atomically sharp interface.

To confirm that the exciton–SPP–exciton conversion process is indeed responsible for the observed phenomena, we performed two control experiments. First, the excitation laser was moved to a region of the Ag plate without any TMDs. Neither propagating SPP nor PL signal was detected, as shown in Fig. 2e. These experiments confirm the high surface quality of Ag plate; incident plane waves cannot excite SPP modes on a planar metal/dielectric interface due to the momentum mismatch while SPP modes on a rough metallic surface can be directly excited. In other words, optical excitation in the WS_2_ region indeed induces the point sources necessary to launch the SPPs in the investigated MOS hybrid platform. We then mapped PL from a TMD heterostructure on a Si/SiO_2_ substrate with a fixed excitation location, as shown in Fig. 2f. No PL signal from other regions of the sample was collected except for at the excitation spot, indicating that the exciton diffusion length is within the spot size. This experiment demonstrates that long-distance energy transfer can only be realized in the hybrid MOS platform.

### Energy transfer distance

**Fig. 3:**
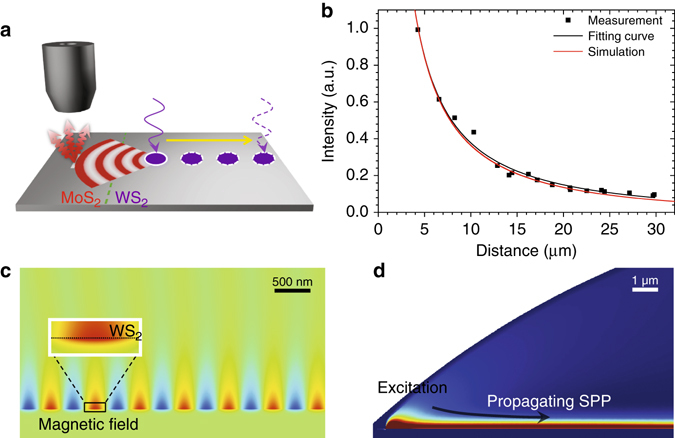
Measurements and simulations of exciton energy propagation process. **a** Schematic of measurement geometry for determining the exciton energy propagation length. The PL intensity is collected at a fixed location on the MoS_2_ region, beneath which a groove is fabricated on the Ag plate. The excitation laser spot is moved away from the groove during measurements. **b** Measured PL intensity (*black points*) vs. increasing distance between the collection and launching sites. Two curves correspond to a fitting result (*black curve*) and the simulated energy decay behaviour in the MOS structure (*red curve*). **c** Simulated near-field profile suggests that a significant percentage of the transferred energy is distributed outside of the TMD LHS monolayer. **d** Simulated power-density profile and the decay behavior along the propagation direction

Next, we quantify the propagation length associated with the SPP-assisted exciton energy transfer process. A series of PL spectra have been collected at the same position around the out-coupling groove while the excitation laser spot was moved away from the collection spot (Fig. 3a). In Fig. 3b, the integrated PL intensity is plotted as a function of the distance between the launching site on WS_2_ and the collection site on MoS_2_. Full-wave simulations (see Methods for details), employing experimentally measured optical constants of crystalline Ag^27^ and WS_2_^28^, predict ~34 μm decay length in the presence of the WS_2_ layer, as shown with solid redline in Fig. 3b. The magnetic field (snapshot in time) and power-density distribution of the SPP wave are shown in Figs. 3c, d. Noticeably, the SPP wave is significantly extended in air (upper region of the surface waveguide), resulting in large propagation distance of the mode, in spite of the moderately large absorption in WS_2_ and Ag. The propagation distance also strongly depends on the distance between the absorbing WS_2_ layer to the Ag and oxide interface, where the field intensity is maximal. In the present MOS structure, the WS_2_ is very close to the metal-dielectric interface (~3 nm). Thus, a very thin layer of WS_2_ reduces the propagation distance by a factor of three from ~100 μm in the bare plasmonic waveguide to ~34 μm in the MOS structure. The measured data is fitted to an analytical expression that takes into account energy transport in a 2D plane (see Methods). The fitting yielded a propagation length of 43 μm. The remaining discrepancy between the experimentally extracted energy propagation decay length and the simulated one likely arises from the uncertainty in the oxide layer thickness (2–4 nm) and the sensitivity of the fitting to the noise in the PL intensity, especially for the first few data points.

## Discussion

The current experiments on the hybrid MOS with unstructured Ag plates have not been designed to optimize the efficiency in the exciton**–**SPP**–**exciton conversion process, but instead to demonstrate the cascaded exciton transfer process mediated by SPP. Engineering local light-matter interaction via antennas, at the excitation or detection sites, might allow us to improve the energy conversion efficiency^29, 30^. However, such structures will introduce ambiguity if excitons are really involved in the excitation or detection step, since SPPs can be directly excited by laser or out-coupled via antennas. Similarly, the cascaded exciton energy transfer can only be demonstrated conceptually in TMD LHS. If only one TMD material is present, scattered SPPs rather than PL from excitons would dominate the out-coupled signal. In contrast, the energy down-conversion in LHS allows one to distinguish the exciton emission from the out-coupled SPPs at the collection site.

In conclusion, we have demonstrated that the energy of excitons, excited in one material region of a monolayer TMD LHS, can be transferred to another material region by coupling to the SPPs in an MOS hybrid material platform with the plasmonic substrate essentially acting as a planar waveguide. The planar MOS geometry used in our work has several advantages. First, the geometry is extremely simple, and no nanofabrication process is necessary to realize 2D exciton energy transfer. Second, the plasmonic substrate supporting a large optical density of states harvests the exciton emissions with high efficiency and enhances the emission intensity. Our experiments show that the atomically sharp interface in high-quality TMD LHS does not present as an energy transfer barrier. Combined with versatile methods recently developed to control light–matter interaction using plasmonic metasurfaces at the subdiffraction length scale, our work will stimulate further developments incorporating the newly created atomically thin semiconductor heterostructures for a wide range of applications including optical signal processing and photovoltaics.

## Methods

### Growth of TMD LHS

Two representative lateral WS_2_/MoS_2_ heterostructures were respectively obtained with the one-step synthesis using atmospheric pressure CVD (APCVD) and two-step synthesis by combining APCVD in the first growth step and low pressure CVD in the second step. Compositional alloying in the multi-elements CVD synthesis could be avoided with a low temperature CVD synthesis using perylene-3,4,9,10-tetracarboxylic acid tetrapotassium saltas the seeding promoter. The monolayer TMD LHS were synthesized at 650 °C for 5 min with a heating rate of 15 °C/min and a mixed Ar/H_2_ gas flow (15/1sccm). In the one-step synthesis, homemade quartz reactors were used to precisely control the gas flow and the reactions of starting reactants in an one-inch tube furnace growth system.

### Synthesis of colloidal Ag plates

The single-crystalline Ag plates were synthesized using a platinum (Pt)-nanoparticle-catalyzed and ammonium hydroxide (NH_4_OH)-controlled polyol reduction method^17^. The synthesis was conducted at room temperature in a chemical solution.

### TEM and STEM-HAADF

Field-emission transmission electron microscope (TEM, JEOL, operated at 200 kV with a point-to-point resolution of 0.2 nm) was used in this work. Monolayer LHS samples were transferred with a conventional polymethyl methacrylate-assisted technique to Cu grids for TEM and aberration corrected STEM high-angle annular dark-field (STEM-HAADF) imaging.

### ALD of Al_2_O_3_ layer

ALD of high-quality Al_2_O_3_ was grown as a protection layer on the surfaces of Ag to avoid oxidation of Ag plate during the optical measurements. After the synthesis of Ag plate, the samples are immediately capped with a 3-nm-thick Al_2_O_3_ in the ALD chamber to preserve the sample quality.

### Optical measurements

**Fig. 4:**
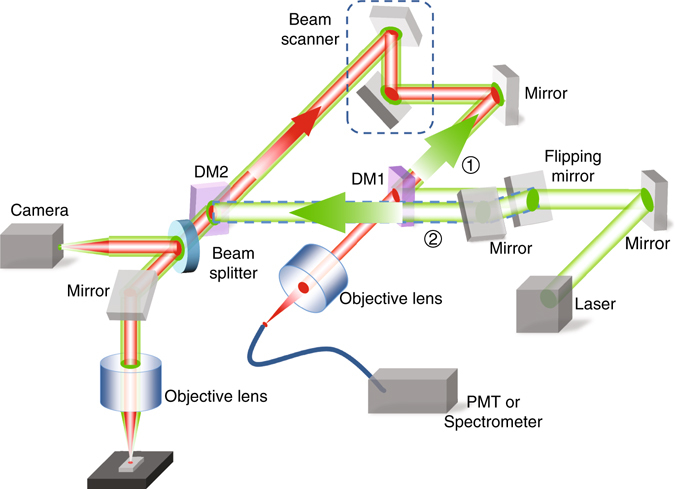
Illustration of the optical measurement set-up. The optical path following the excitation laser is shown in *green* and that of the collected PL signal is shown in *red*. Flipping mirror switches the excitation beam to either (1) double scan mode or (2) single scan mode. DM1 and DM2: dichromatic mirrors

The PL measurements were performed with a home-built laser scanning confocal microscope system, as shown in Fig. 4. There are two operational modes (single-scan and double-scan modes), which can be switched by a flipping mirror. In the double-scan mode, both the laser excitation spot and PL collection location are scanned simultaneously, i.e., the excitation spot (~1 μm in diameter) and PL are from the same region of the sample. In the single scan mode, the excitation laser is fixed at a specific location. The location from which the PL is collected is raster scanned across the sample, i.e., PL from locations different from the excitation spot is recorded. This single scan mode was used for all experiments reported here, aiming to demonstrate exciton energy transfer in the MOS structure.

### Curve fitting

The decay of the PL intensity is fitted with the following equation:

1 $$I = {I_{\rm{b}}} + {I_0}\frac{{{{\rm e}^{ - x/ {\it {\Lambda }}}}}}{x},$$

where *I*_b_ is the background signal, *I*_0_ represents the initial intensity at the launching site and *Λ* is the propagation length (or decay length) associated with the energy transfer process. In addition to the exponential decay due to SPP loss, the *x* ^−1^ dependence arises from the fact that the SPPs propagate in all directions in a 2D plane. *Λ* is found to be ~43 μm in our experiment. The experimental data shown in Fig. 3 is normalized to the PL intensity at the launching site:

2 $$I = \frac{{{\int} {\left( {{I_{{\rm s}{\rm i}{\rm g}}} - {I_{{\rm b}{\rm g}}}} \right)} }}{{{\int} {\left( {{I_{{\rm l}{\rm a}{\rm u}{\rm n}{\rm c}{\rm h}}} - {I_{{\rm b}{\rm g}}}} \right)} }},$$

where *I*_sig_, *I*_bg_ and *I*_launch_ are PL intensities at the collection site, background and PL intensity at the launching site, respectively. As seen from Fig. 2f, *I*_sig_ contains both the MoS_2_ exciton PL component and the out-coupled SPP. We collected PL from the same location while moving the excitation to ensure that the relative intensity of these two components (i.e., exciton PL and out-coupled SPP) remained the same for all measurements. Because of the fixed ratio between these two components, using the total output intensity to extract the energy transfer distance is equivalent to using the MoS_2_ PL intensity for this purpose.

### Simulations

The exciton-SPP coupling and SPP characterization were numerically studied through full-wave simulations in COMSOL Multiphysics in the frequency domain using radio frequency module at 630 nm. The structure was modeled as a 2D multilayer waveguide excited with an electric dipole positioned inside the WS_2_ layer. As shown in Fig. 3, the SPP mode generated at the Ag-dielectric-WS_2_ interface is significantly extended in air (with an exponential decay), lying close to the light line. Power distribution in the proximity of the excitation is illustrated in Fig. 3d. In order to avoid near-field effects close to the excitation point, power intensity is sampled with at least 20 μm lateral distance from the excitation to calculate the propagation distance. In the 2D model, an exponential decay along the propagation direction was used to extract the propagation length, which is then used to produce *red curve* in Fig. 3b.

The WS_2_ layer is modeled as a sheet admittance^31^:

3 $$Y = \frac{{j{k_0}t\left( {{\varepsilon _{{{\rm W}{\rm S}}_2}} - 1} \right)}}{{{\eta _0}}},$$

where *Y* is the admittance, ${\varepsilon _{{\rm{W}}{{\rm{S}}_2}}}$ is the permittivity of the material and *t*=0.81 nm is the layer thickness. *k*_0_ and *η*_0_ are free-space wavenumber and intrinsic impedance, respectively.

The simulation domain is a circular segment (Fig. 4b) truncated with a perfectly matched layer at the top, and a 400-nm-thick grounded silver layer at the bottom. The thickness of the oxide layers on both sides of theWS_2_ are estimated as 3 nm from fabrication process. Maximum mesh sizes of 5 nm and 15 nm are used for oxide layers and silver substrate, respectively. The air domain is meshed with maximum element size of 15 nm in the proximity of the WS_2_ and 80 nm in the far-field. The propagation distance is calculated from the SPP power sampled 100 nm above the silver interface.

### Data availability

The data that support the findings of this study are available from the corresponding author upon reasonable request.

## Electronic supplementary material

Supplementary Information Supplementary Figures, Supplementary Tables, Supplementary Notes and Supplementary References
