# Supplementary material for: Cascaded exciton energy transfer in a monolayer semiconductor lateral heterostructure assisted by surface plasmon polariton
Source: Nat Commun. 2017 Jun 26;8:35. doi: 10.1038/s41467-017-00048-y (PMC5484701; doi:10.1038/s41467-017-00048-y)
Supplement: Supplementary file 1 — Supplementary Figures, Supplementary Tables, Supplementary Notes and Supplementary References [file 41467_2017_48_MOESM1_ESM.pdf]

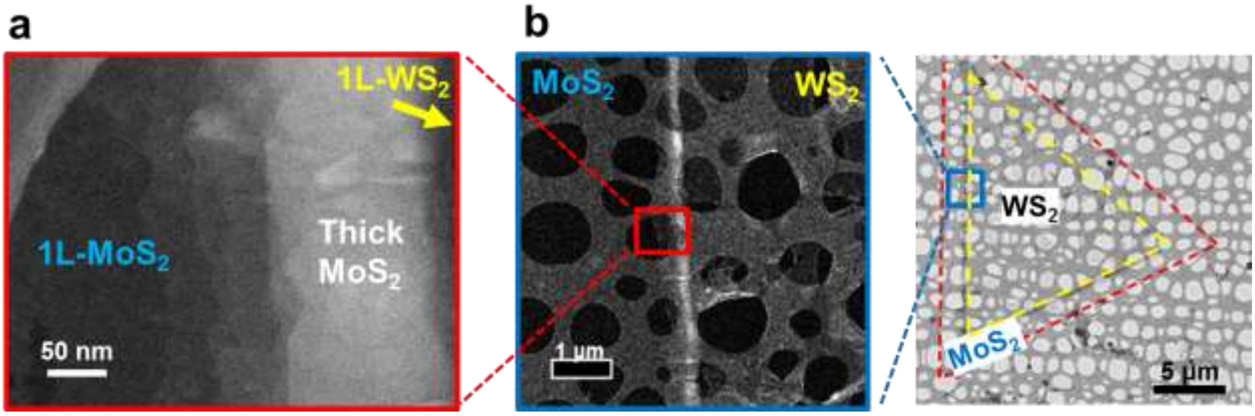

Supplementary Figure 1. TEM image of the lateral WS_2-MoS_2 heterojunction with "rough" interface. a High-resolution TEM image indicates stacks of MoS_2 formed at the interface area. **b** Low magnification TEM image clearly shows that MoS_2 stacks are distributed over the entire heterojunction. The yellow dashed lines denote the boundary between WS_2-MoS_2.

Supplementary Note 1

The rough interface of a WS_2-MoS_2 lateral heterostructure (LHS) grown by the two-step growth process is investigated via transmission electron microscope (TEM) as shown in Supplementary Figure. 1. Stacks of small MoS_2 flakes are formed over the entire heterojunction. The number of stacks varies depending on details of the growth conditions and it can be tens of layers.
